# Forget-me-some: General versus special purpose models in a hierarchical probabilistic task

**DOI:** 10.1371/journal.pone.0205974

**Published:** 2018-10-22

**Authors:** Franziska Bröker, Louise Marshall, Sven Bestmann, Peter Dayan

**Affiliations:** 1 Gatsby Computational Neuroscience Unit, University College London, London, United Kingdom; 2 Department for Movement and Clinical Neurosciences, UCL Queen Square Institute of Neurology, University College London, London, United Kingdom; Brain and Spine Institute (ICM), FRANCE

## Abstract

Humans build models of their environments and act according to what they have learnt. In simple experimental environments, such model-based behaviour is often well accounted for as if subjects are ideal Bayesian observers. However, more complex probabilistic tasks require more sophisticated forms of inference that are sufficiently computationally and statistically taxing as to demand approximation. Here, we study properties of two approximation schemes in the context of a serial reaction time task in which stimuli were generated from a hierarchical Markov chain. One, pre-existing, scheme was a generically powerful variational method for hierarchical inference which has recently become popular as an account of psychological and neural data across a wide swathe of probabilistic tasks. A second, novel, scheme was more specifically tailored to the task at hand. We show that the latter model fit significantly better than the former. This suggests that our subjects were sensitive to many of the particular constraints of a complex behavioural task. Further, the tailored model provided a different perspective on the effects of cholinergic manipulations in the task. Neither model fit the behaviour on more complex contingencies that competently. These results illustrate the benefits and challenges that come with the general and special purpose modelling approaches and raise important questions of how they can advance our current understanding of learning mechanisms in the brain.

## 1 Introduction

Humans are remarkably good at adapting to statistical structure in their environment by efficiently turning stochastic observations into both actions and expectations over future experiences. In simple cases, such as enduringly popular forms of probabilistic binary sequences, predictions appear to be consistent with the precepts of optimal Bayesian reasoning [1–6]. Though such feats are impressive, their very optimality makes it hard to probe the underlying mechanisms, since algorithms are usually revealed by their idiosyncratic flaws.

When sequences become more complicated, optimal methods become computationally challenging, and more substantial and revealing approximations can arise. Previous work [7–9] suggests that this may be because subjects do not recover the true models of the tasks if they display some level of complexity. Thus, ideal observer models assuming knowledge of the true model and optimal learning within it fail in these contexts. Indeed, learning the structure of the task is a difficult problem [10–12], and it appears more likely that humans employ good approximate solutions. In the realm of such nearly normative characterisations, a particular distinction arises between assuming that subjects employ rather powerful generic reasoning models which then require potent approximation methods, versus assuming more narrowly specific, simplified, models that are tailored to the problems at hand.

Generic models are appealing because they offer a unified framework that can be applied to a variety of learning conditions. Subjects might apply them as a default when first required to perform a task. More tactically, from the perspective of experimenters, they make it straightforward to compare and interpret results across datasets and tasks. However, generalisability usually comes at the cost of accuracy. Thus, we can expect subjects to be able to tune their inferential apparatuses to particular tasks, capturing aspects that general approximation schemes miss. To the extent that this is the case, it becomes important to interrogate closely conclusions drawn on the basis of generic models.

In this paper, we examine in detail a test case for this contrast coming from a richly complex sequential problem administered by some of the current authors [13]. Subjects carried out a probabilistic serial reaction time task (pSRTT) involving three particular dimensions of complexity: four stimuli rather than the more conventional two [14–17]; Markov rather than only memoryless sequential dependencies [16, 17]; and unsignalled changes in these dependencies [18, 19]. Behaviour was examined under placebo, and under antagonists of the neuromodulators acetylcholine (ACh), noradrenaline (NA) and dopamine (DA); the former two of which have been implicated in reporting forms of uncertainty associated with learning [20, 21]; and the last in exerting influence over response vigour or speed [16, 22–26].

Previous studies of learning in hierarchical tasks manipulating the stability of the environment have shown that ideal observer models fail to accurately account for subjects’ behaviour. Summerfield et al. [8] observe that hierarchical structure was not recovered by subjects in environments being highly volatile, and that optimal inference only occurred over periods of great stability. Similarly, Norton et al. [9] reported that a model with simple exponential weighing of experience can outperform an ideal Bayesian learner in static and dynamic environments. Furthermore, Fusi et al. [7] identified that monkeys show trial-and-error behaviour after cue-reversals before undergoing slow relearning of the association showing that the animals did not learn to adapt optimally to the switching process. This strongly suggests that models employing approximations will be necessary to account for the data from our complex pSRTT too.

In the previous work, it was assumed that subjects’ behaviour would be best described by a paradigmatic generic method called the Hierarchical Gaussian Filter, HGF; [27]. This model is a predictive coding model [28], and has been applied to a wealth of different problems [13, 29–36]. The parameters of the HGF were fit in placebo and drug conditions and systematic differences were examined.

Here, we address the question of whether a more specific, non-general model could capture subjects’ behaviour more proficiently. After describing the task, we use a subset of the pSRTT data, namely simple sequences in which the appearance of one symbol was independent of the previously observed symbol (memoryless or 0^th^-order sequence), to show lacunæ in the HGF’s characterisation of subjects’ behaviour. Then, we consider a simple, specific model (the Forgetful Observer Model; FOM; [37]), which assumes that subjects used limited memory to adjust to environmental requirements on the fly rather than capturing them with successively higher hierarchical levels of a probabilistic characterisation. This simpler model turns out to fit significantly better. We then use the new account to reanalyse the behaviour under the various pharmacological conditions. Finally, we turn to the more complex temporal sequences.

In sum, we demonstrate some of the difficulties of making inferences about learning in hierarchical tasks and discuss the benefits and challenges of general and special purpose models in the light of these results.

## 2 Methods

### 2.1 The task

We characterise the results of a probabilistic serial reaction time task (pSRTT) that had been designed to investigate online learning under multiple forms of uncertainty. Full details of the experimental procedures are available in [13].

In brief, subjects saw a sequence of four different symbols, each of which was associated with its own required keypress according to a mapping that subjects had previously learned. Stimuli were presented for 200 ms, followed by an interstimulus interval (ITI) of 1200 ms. Missing responses and anticipatory button presses (< 80 ms) were recorded as being incorrect.

**Fig 1:**
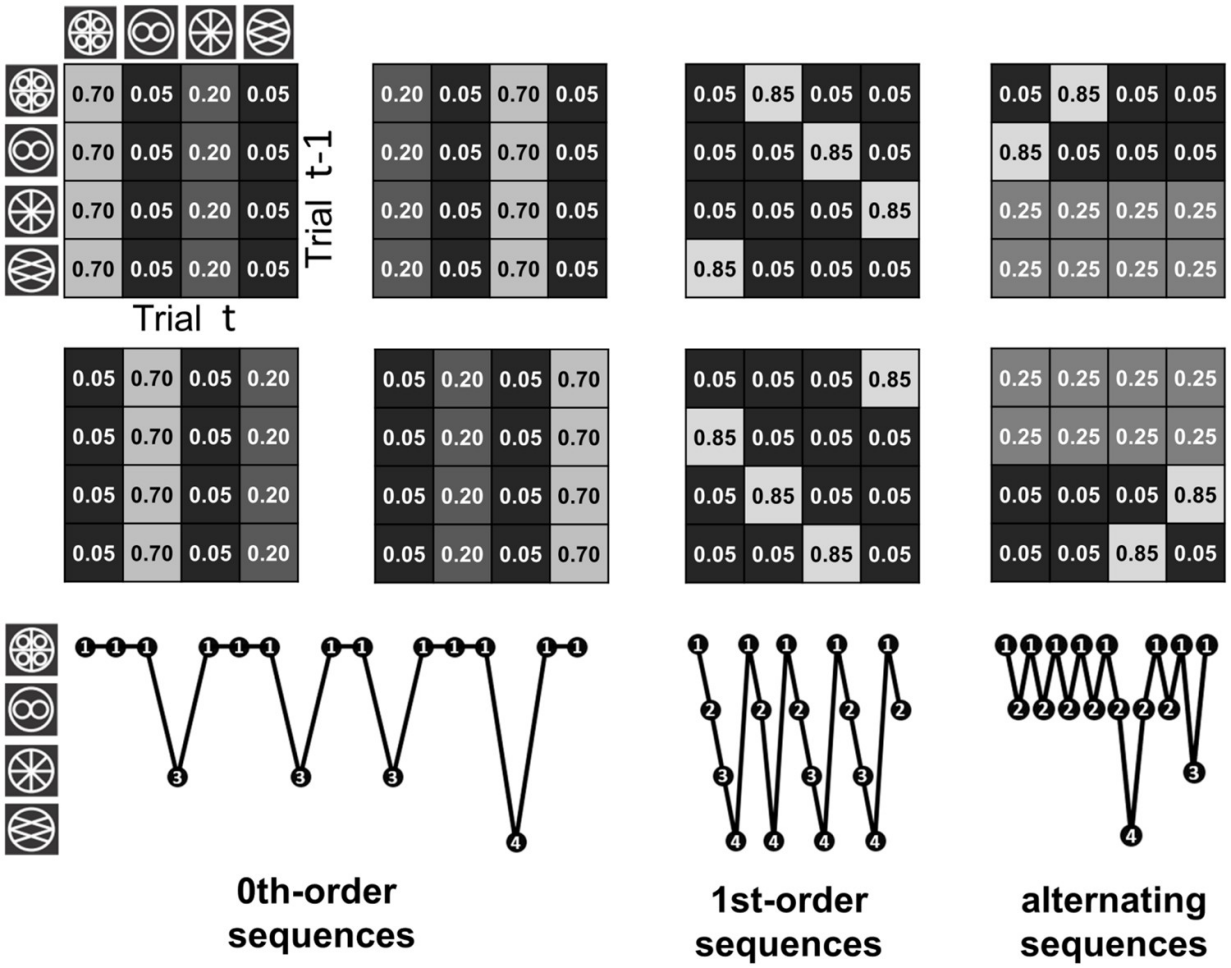
Transition matrices T’s used to generate stimulus sequences within contextual blocks. Over the course of the experiment, four 0^th^-order sequences, two 1^st^-order sequences and two alternating sequences occurred, three times each. Here, as is conventional for Markov chains, we show each $T_{ij}$ as *p*(*s*^*t*^ = *j*|*s*^*t*−1^ = *i*) (which is the transpose of the way the transitions were shown in [13]). The different dynamics of the matrices are illustrated by example sequences generated from each of the three matrix types (i.e. from matrices 1, 3 and 4 in the upper row).

The order in which the symbols were presented reflected a probabilistic dependency structure involving two hierarchical levels. At the lower level were blocks of 50 trials, each associated with a simple Markov chain. That is, the symbol sequence in a block was generated in a pseudo-random order from a 4 × 4 transition matrix T determining the transition probability from one stimulus to the next (Fig 1). There were three classes of T, characterised by 0^th^-order (4 matrices), 1^st^-order (2 matrices) or alternating dynamics (2 matrices); Fig 1.

At the upper level of the probabilistic structure, the eight Markov chains were presented three times each in a pseudo random order (without any immediate repeat). The probabilistic relationships between stimuli in successive trials and changes between blocks were unknown to the subjects.

Finally, subjects were randomly allocated in a double-blind design to one of four experimental groups, receiving a placebo or an antagonist of either DA, ACh or NA in order to assess the effects of the respective neuromodulator on the inference process. Data from a total of 124 subjects were analysed.

### 2.2 Model fitting

In this paper, we report the results of fitting two different classes of models to the data of the subjects. The different types of transition matrix lead to characteristically different results for both models. Therefore, we evaluate the models in two steps. For the bulk of the analyses, we fit the models to the entire sequence of data, but place a special emphasis on qualitative and quantitative fits of only the data coming from the the simplest and most prevalent matrix type: the 0^th^-order sequences. In section 3.5, we separately fit our model to the sequences from the different matrix types in order to validate the conclusions drawn from the full model and to highlight the difficulties of modelling higher-order matrices. For these models we assume that the statistics associated with the transitions were reset at the beginning of each block. This is purely for illustrative purposes—the subjects received no direct information about block boundaries.

The models were fit in Matlab R2015a and were compared according to their BIC values (the lower the better), which trade off model fit (likelihood of the data) and model complexity (number of fitted parameters).

## 3 Results

We fit two characteristic trial-by-trial learning models to subjects’ behaviour. The models differ in their assumptions about the underlying mechanisms of learning. The first model, the Hierarchical Gaussian Filter (HGF; [27, 38]), was used by Marshall et al. [13] in a first treatment of the data. The HGF is designed to generalise over a variety of learning tasks, and so starts minimally fashioned to the actual task at hand. This inevitably limits its ability to encompass the data for particular tasks, and motivates an alternative, forgetful observer model (FOM; [37]), that is more closely adapted to the task.

Both models have two components: a *perceptual component* that captures the latent or unobservable inference process assumed to be employed by the subject to produce a probability distribution over the next symbol; and a *response component* that turns this distribution, plus features of past responses, into a distribution over the single, observable, quantity, namely the time it takes the subject to make the key press [39]. We fit these components jointly, and thus infer states and parameters in the proposed perceptual components. We consider qualitative and quantitative features of the fits of the two models.

After analysing 0^th^-order sequences in detail, we more briefly discuss the rather substantial underfitting of both models to the higher order transition matrices.

### 3.1 Hierarchical Gaussian Filter

The Hierarchical Gaussian Filter (HGF, [27, 38]) is a recursive filter that approximates Bayesian recognition for a hierarchical generative model. It casts online learning as a broadly applicable inference algorithm [29–36] and requires attractively little adjustment for a particular task environment. Learning about the statistical structure of the environment is characterised as a hierarchical inference scheme in which each level is modelled by a Gaussian random walk. In the generative aspect of this scheme, the state at one level determines the lability of the level below. Thus, in the recognition aspect of the scheme, which underpins the perceptual model, the chains are coupled such that the learning rate at one level is influenced by the state at the next higher level. According to an approximate variational inference scheme, precision-weighted prediction errors propagate between levels to modify states.

In the previous work, a three level version of the HGF was employed as the perceptual component. The lowest level of the model involved a collection of 4 × 4 = 16 independent Gaussian random walks governing the propensity of each of the 16 possible transitions. The middle level was a set of evolving 4 × 4 = 16 numbers governing these transition contingencies. Finally, the top level captured the volatility of the latter dependencies. In the response component of the model, RTs were predicted from a linear combination of a model-agnostic variable (the effect of coming straight after an error trial), and model-dependent variables, including precision-weighted prediction errors and volatility.

It is important to note that the full formulation of the HGF itself is not limited to the particular version discussed here. However, for convenience, in the following, we will refer to the particular treatment in [13] as just ‘the’ HGF.

This version of the HGF had a total of 10 subject-specific parameters: parameters of the prior over the initial state of volatility ($\mu_{3}^{\left(0\right)}$ and $\sigma_{3}^{\left(0\right)}$), a metavolitility parameter controlling the step size of the Gaussian walk at the third level (*ϑ*), a tonic learning parameter controlling the step size of Gaussian walks at the second level (*ω*), coefficients in the response component (*β*_0−4_) and the response variance in the response component (*ζ*). Additional restrictions on the parameter space were introduced by a pre-specified upper bound on *ϑ*, priors on the response coefficients, and a strong prior on *ζ*; we consider the effects of these below.

The model was fit to the RTs of each subject in a stepwise manner. First, the perceptual component was fit to the actual sequence of symbols presented (i.e., independent of what a subject did) to create prior distributions over the parameters consistent with approximately optimal inference. Prior means for parameters in the subsequent data analysis were obtained from these optimal posterior estimates and prior variances were specified by hand. Then, the parameters of the perceptual and response components were jointly fit to the RTs using these priors for the former, and the restrictions mentioned above. The effects of drugs were estimated in a between-subjects manner by assessing the parameters for each group of subjects, taking body weight and alertness into account via a linear model. A recovery analysis was performed for the parameters controlling the step size of the Gaussians on the second (*ω*) and third level (*ϑ*) of the HGF’s perceptual component. The results show that the former was well recovered while the estimates of the metavolitility showed substantial deviations from the true parameter value limiting the interpretability of quantities on the top level of the HGF (see S1 Fig).

In terms of the present study, the HGF was inherently approximate as a generative model. First, discrete changes (between T’s) were modelled by continuously evolving Gaussian random walks, thus eliminating actual transitions. Second, the estimates of the elements of a well-founded transition matrix T were replaced by independent Gaussian random walks that were not normalised by starting state (i.e., by row). Amongst other consequences, this implies a lack of competition between the different possible symbols. Equivalently, subjects were implicitly assumed to be allowing more than one symbol to appear on a trial. Inference in the HGF, which is variational, was also an approximation to full Bayesian calculations.

**Fig 2:**
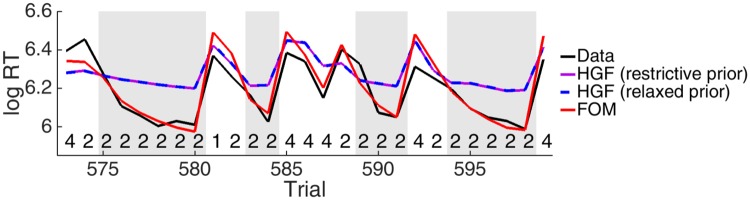
Example sequences for the 0^th^-order sequences: Experimental data (black), and predictions for the original (purple) and relaxed HGF (blue) and the FOM (red) averaged over subjects in the placebo group. High probability transitions from stimulus 2 to 2 are shaded in grey. The stimulus sequence is shown underneath the lines.

Fig 2 provides a representative illustration of the power and problems of the HGF. This shows the actual and modelled RTs averaged over subjects in the placebo group during an example sequence of symbols during a 0^th^-order matrix (for which symbol 2 is most common). The black line shows average data from the placebo group. It is apparent that the subjects greatly increased their speed over periods in which only the high probability symbols were shown (shaded in grey), and then slowed down markedly for every lower probability stimulus.

By contrast, the purple line shows the result of fitting the HGF to these data, again averaged across predictions for individual subjects in the placebo group. It is apparent that the HGF failed to capture the large degree of modulation during the epochs of higher probability, although it was appropriately affected by the low probability interjections.

As mentioned above, a restrictive prior was specified on the variance *ζ* of the response component of the HGF in order to strengthen the contribution of the data during model optimisation. However, this narrowed the distribution predicted by the model so much that log likelihoods could not be compared to models with unaffected variance estimates. Thus, for our purposes, we fitted the HGF with a relaxed prior (ζ∼N(-3,100)), which allowed a wider predictive distribution and consequently a substantially increased log likelihood (LL; Table 1). Since the fit to the data remained essentially identical (shown by the blue line in Fig 2) and the same conclusions about differences in the parameters of the groups could be drawn from this model, we based all further analyses and comparisons on this adjusted HGF.

**Table 1:** Model summary. Statistical models with number of parameters per subject, average log likelihood and BIC value. Details on the fit for the different drug groups are given for the favoured HGF and FOM. These values concern all the sequences of trials.

Model	Parameters	average LL ×10^4^	BIC
HGF (restrictive prior)	10	65	6936
**HGF (relaxed prior)**	**10**	**218**	**2888**
Placebo		68	1766
DA		-50	2578
ACh		785	-2769
NA		129	1313
FOM (stable beliefs, *γ* = 0)	4	84	1234
**FOM** (**converging beliefs**, *γ* = 1)	**4**	**602**	**-12469**
Placebo		458	-2277
DA		267	-938
ACh		1233	-6714
NA		518	-2540
FOM (unconstrained beliefs)	4	584	-11979

The dependence between the goodness of fit measure and the (hand-crafted) prior is exceptionally severe in this case. This is because a prior on the response variance directly influences the reference distribution to which the data are compared as implemented in the log likelihood measure, and consequently the BIC value. However, it should be noted that the impact of priors on the log likelihood score does not transfer to the rest of the HGF’s priors which affect the posterior response distribution less directly.

**Fig 3:**
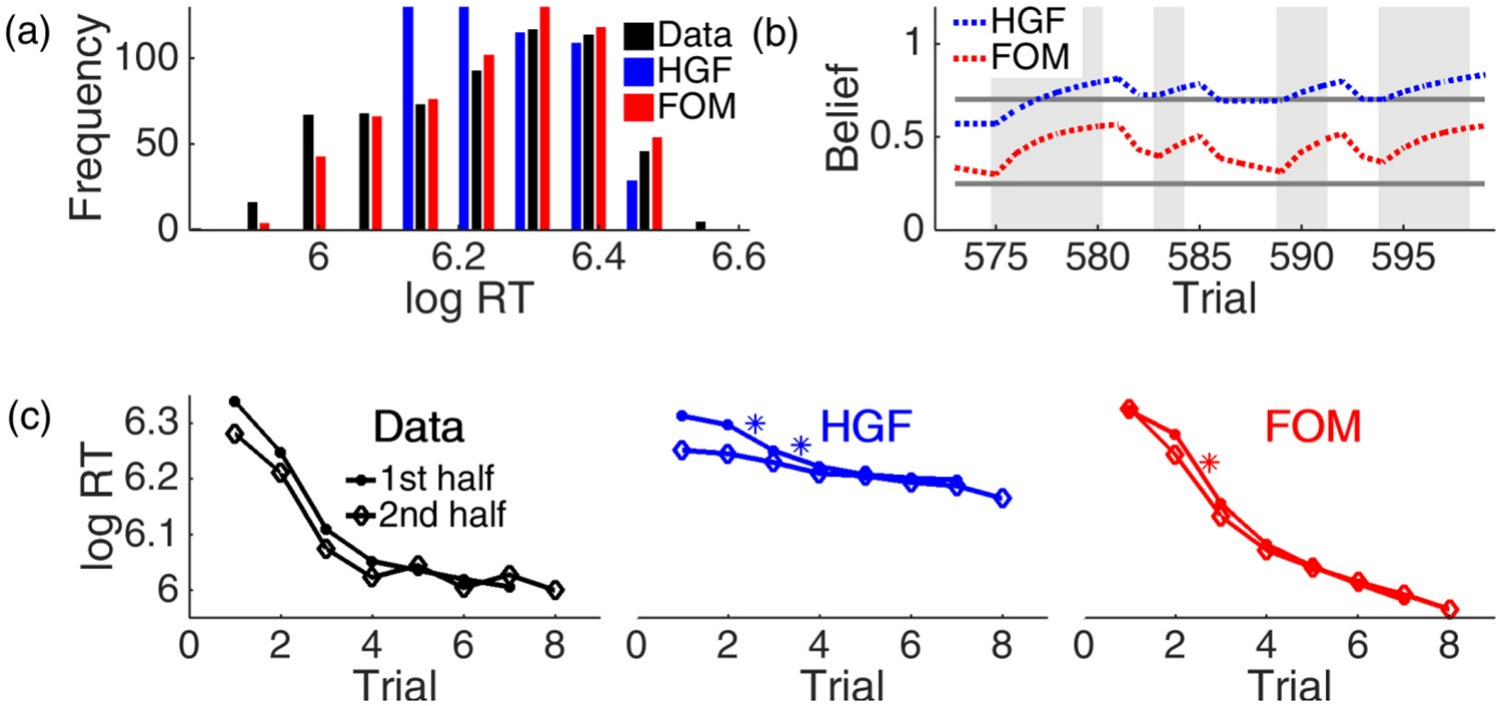
Model fits to the 0^th^-order sequences. RTs in the placebo group are shown in black and predictions of the HGF in blue and FOM in red. (a) Distribution of average RTs and predictions. (b) Estimates of the probability for transition from stimulus 2 to 2 (shaded grey) in the sequence shown in Fig 2 simulated from the average parameters inferred from the placebo group. The horizontal lines indicate uniform belief (0.25) and the transition probability of the predominant transition (0.7) of the transition from stimulus 2 to the next stimulus. (c) RTs and model predictions averaged over all uninterrupted sequences of the high probability stimulus separated according to whether they started in the first or second half of blocks. Trials on which the speeding curve was significantly shallower during the second half compared to the first are marked by asterisks.

Fig 3 shows the problems of the HGF fit in three different ways. First, Fig 3a shows a histogram representation of the full distribution of predicted (blue) and actual (black) RTs. The underestimation of the way the subjects sped up is apparent as a compression in the range, with an overly frequent prediction of moderate RTs.

Second, Fig 3b shows the perceptual model’s inference about the probability of the most frequent transition during the sequence in Fig 2. It is apparent that the HGF learned this probability rather quickly—even exceeding the true probability value of 0.7. However, it was then left unable to fit the successive speeding of the subjects during the high probability periods, as its beliefs did not decline substantially during low probability trials. To put this another way, the HGF underestimated the lability of the subjects’ beliefs, having too large a window of integration. Indeed, it seems that subjects changed their beliefs significantly even within blocks. Thus the subjects readily re-learnt probabilities after unexpected transitions, whereas the HGF did not.

Fig 3c provides a different view of the same issue. This shows the speeding curves averaged over all high probability sequences and separated over the first and second half of the blocks, for the data and the HGF. Again, the HGF appeared insufficiently labile. Fig 3c also suggests another limitation of the HGF. One key characteristic of the task is the discrete changes to the transition matrices T. There are various ways to capture changepoints in Bayesian models [18–20, 40]. However, before fitting these, we conducted some model-agnostic analyses to determine whether subjects showed any sensitivity to the changes. An obvious signature would be that the RTs following high probability transitions in the second half of a block would not only be faster than those in the first half, but, crucially, would also change less over a high probability sequence. That is, the speeding curve in the second half of a block would be shallower. Both of these phenomena would arise because of learning.

We therefore analysed the speeding curves of subjects in the placebo group during in the 0^th^-order blocks. We sought to compare RTs between the first and second halves of blocks on unbroken sequences of the predominant stimulus to investigate any differences in the degree to which subjects increased their speed from one trial to another. Since the speeding curves were somewhat idiosyncratic, we separately compared speeding on successive trials for each trial *t* within a high probability sequence (i.e. comparing (zlogRT^t+1^ − zlogRT^t^) between block halves where zlogRT denote log RTs, and predicted log RTs respectively, that were z-scored for individual subjects with mean and standard deviation based only on the trials from unbroken 0^th^-order sequences). We constructed permutation tests by shuffling between block halves the (zlogRT^t+1^ − zlogRT^t^) within subjects (10000 iterations) for each trial. As expected, the extent by which the subjects sped up on unbroken sequences did not significantly differ between first and second half of a 0^th^-order block (all p-values ≥ 0.2). That is, the speeding curves were parallel.

In contrast, the HGF predicted a significantly shallower speeding curve for responses in the second than the first half of blocks on trials 2-4 (*p*_2,3_ < 0.001, *p*_3,4_ = 0.01). This would have implied that subjects would have been sensitive to the predictive failure consequent on a block change, allowing them to rely in the second half of a block on the knowledge about transitions acquired in the first half.

Thus, the data suggest that the chain of observations were perceived more as coming from an inherently unstable environment with only short periods of predictability, than from a sequence of separable probabilistic contexts (as considered, for instance, in work on unexpected uncertainty; [20]). Even though the HGF lacked the discrete change process which is actually true of the data, its hierarchical structure allows for variable learning rates that provide an approximate alternative model to rapid changes. However, the parameters of the HGF were set into a range (potentially further restricted by the upper bound on the meta-volatility *ϑ*) in which there is insufficient echo of the quickly changing beliefs observed in this task. Indeed even only a few unexpected observations led to substantial slowing, followed by the observed re-learning within contextual blocks. To put it another way, the block structure apparently had less impact on subjects’ learning than predicted. These results echo previous observations that learners fail to exploit the hierarchical nature of volatile environments [7–9].

### 3.2 Forgetful Observer Model

These various findings suggest that the generality of the HGF may have impacted its ability to fit the data. Instead, the fact that subjects’ responses are so labile to changes in the symbols suggests that a model based on ready forgetting, rather than an extra layer of an inferential hierarchy as in the HGF, might fit more accurately. A wealth of models employing the idea that the impact of past experience decays with time have been proposed on different levels of analysis in the past, such as models for neural integration [41–45], computations of neural populations [7], and models accounting for behaviour in tasks of stimulus-response, probabilistic contingency, and reinforcement learning [9, 37, 46–48]. Equally, a model could be built that correctly reflects the subjects’ knowledge that only a single symbol would be presented at any time.

**Fig 4:**
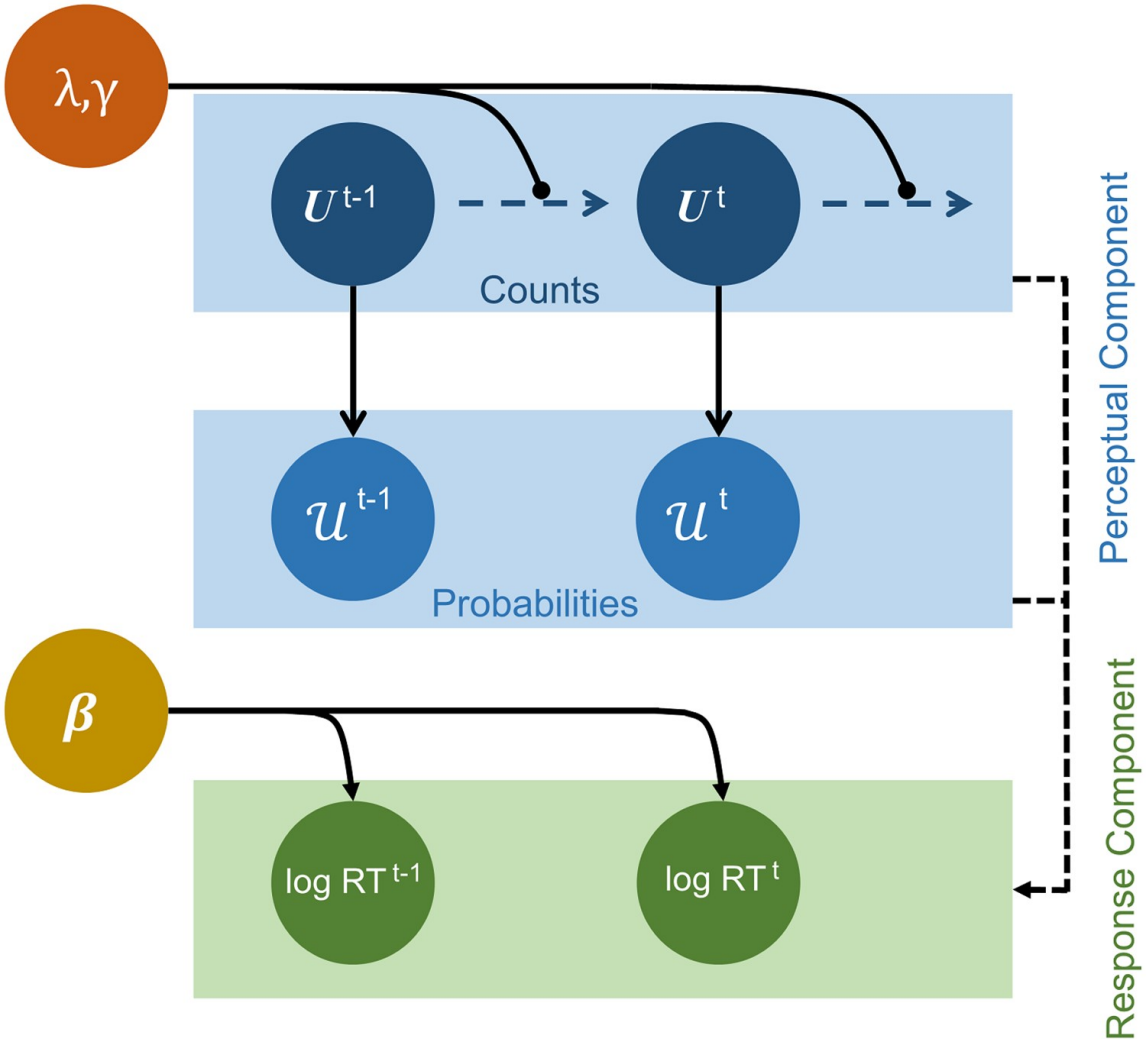
Forgetful Observer Model (FOM). The perceptual component tracks the observers’ learning over just two levels. The lower level represents transition probabilities analogous to the generating transition matrices T’s. The top level represents the parameters of a (forgetful) Dirichlet distribution. These can be interpreted as counting numbers of effective transitions, which are incremented by experience, and decremented to an asymptotic prior *γ* by a forgetting rate λ. The response component predicts subjects’ responses according to a linear combination (with weights ***β***) of quantities in the perceptual component and model-agnostic factors.

Fig 4 shows the perceptual component of the new, forgetful observer model (FOM). While differing in its general mathematical formulation, our specific choices of parameters for this task render the FOM a version of the model suggested by Harrison et al. [37]. The first level of this component is based on the true process that generated the data that the subjects observed. Stimuli (*s* ∈ {1, 2, 3, 4}) are drawn at each trial *t* from a categorical distribution parameterised by transition probabilities ($U_{ij}$ for the transition from stimulus *i* to *j*). This is exactly how the stimuli were actually generated from transition matrix T (Eq 2). Then, at the second level, the rows $U_{i\cdot }$ of U are assumed to be drawn from the conjugate of this categorical distribution, i.e., a Dirichlet distribution. The parameters of the four Dirichlet distributions (*U*_*ij*_) can be interpreted as the number of effective observed transitions. In total, this implies:

$$\begin{array}{c} P\left(U_{i.}^{t}|U_{i.}^{t}\right)=\text{Dirichlet}\left(U_{i.}^{t}\right) \end{array}$$ (1)

$$\begin{array}{c} P\left(s^{t}|s^{t-1}=i\right)=\text{Categorical}\left(U_{i.}^{t}\right) \end{array}$$ (2)

If the environment is known to be constant, then the analytical update equation in this model involves adding counts to the parameter of the Dirichlet distribution that corresponds to the observation. Rather than modelling the details of the process by which the transition matrices actually change (something that the subjects did not know), we adopt the simpler approximate strategy of using a forgetting process as has been used by Harrison et al. [37] to model a similar, 1^st^-order Markov, 4-alternative forced-choice task. This involves two parameters, a forgetting rate λ ∈ [0, 1], which is conceptually related to the tonic learning rate *ω* in the HGF, and an asymptotic prior $\gamma \in R_{\geq 0}$, which is the target of the forgetting process, given no new observation. We discuss the effect of *γ* below.

The update equation for each parameter of the Dirichlet distribution *U*_*ij*_ after observing stimulus *s* at time *t* is then:

$$\begin{array}{c} U_{ij}^{t+1}=\left(1-\lambda \right)(U_{ij}^{t}-\gamma )+\gamma +\delta (\left(s^{t-1},s^{t}\right),\left(i,j\right)) \end{array}$$ (3)

where *δ* is the Kronecker-delta (*δ*(*a*, *b*) = 1 iff *a* = *b*). The Dirichlet distribution is initialised as $U_{ij}^{\left(0\right)}=\gamma$.

The coupling between perceptual and response components was then closely related to that for the HGF. We characterised log RTs as being linearly related to a model-agnostic term (quantifying the effect of post-error slowing) and model-dependent quantities (the probabilistic surprise at the symbol). By comparison with the HGF, we used a flat prior over these parameters since there was no evidence for any specific parameter values prior to this study. In particular, no informative prior was placed over the response variance (which underlay problems in model comparison with the HGF).

According to the model:

$$\begin{array}{c} \log{\left(RT\right)}^{t}=\beta_{0}+\beta_{1}{\bar{U}}_{s^{t-1}s^{t}}^{t}+\beta_{2}\chi \left(e^{t-1}\right) \end{array}$$ (4)

where ${\bar{U}}_{s^{t-1}s^{t}}^{t}$ is the mean of the posterior distribution for the actual transition from *s*^*t*−1^ to *s*^*t*^, and *χ*(*e*^*t*−1^) is 1 if the subject pressed the wrong button on trial *t* − 1 or 0 otherwise. The forgetting rate was optimised using the Matlab function fminsearch, while regression coefficients for the response component of the model were obtained from standard least squares. We validated this procedure by showing excellent parameter recovery based on data generated from the model (see S1 Fig).

Under this model, parameter *γ* has a qualitative effect on the predictions made by the perceptual component, as well as a quantitative one. If *γ* = 0, forgetting is characterised by an increase in uncertainty (coming from the diminishing counts), but stable relative belief estimates (as the ratio of the counts remains stable). Stable relative beliefs are also inherent to the HGF, and this results in qualitatively similar belief trajectories (not shown). However, if *γ* > 0, then beliefs instead asymptote towards uniform expectations about all possible transitions. This allows the model to re-learn transition probabilities continually, not only after context changes but equally strongly within blocks as information from previous trials in a block depreciates.

Figs 2 and 3 show that the FOM could capture the 0^th^-order sequences far more proficiently than the HGF. It provided a particularly good fit for the speeding during the high probability periods. Nevertheless, Fig 3c shows that the FOM predicts significantly shallower speeding from trial 2 to 3 during the second half of a block (*p* = 0.01); while all other comparisons were statistically insignificant.

In the HGF, the deviation from the data arises from its overconfident beliefs. By contrast, in the FOM, the deviation arises from the forgetting process: Since transitions from rare stimuli to the predominant stimulus initialising each high probability sequence are forgotten rapidly, the FOM predicts similar RTs on the first trial of each sequence, wherever it appears in a block, whereas the data shows a clear offset between those RTs in the first versus second half of a block (see Fig 3c at Trial 1). Consequently, the speeding curve predicted by the FOM optimised on the full data is slightly differently scaled in the first and second block.

### 3.3 Model comparison

In order to put the FOM on an equal footing with the HGF for the sake of comparison, individual values of λ and ***β*** were fit to the RT data for each subject. By contrast, *γ* was fixed to 1 for all subjects as in [37] since BIC values did not favour an extra parameter spent per subject, but provided compelling evidence for *γ* > 0 (as discussed below). Note that while the FOM is more general in its hierarchical structure and the additional parameter for the asymptotic prior, the choice of belief measure and value of *γ* renders its behaviour essentially identical to that implied by the exponential forgetting kernel in [37].

The placebo group forgot at an average rate of λ = 0.303 ± 0.222. Furthermore, across this group, all the *β* parameters of the response model were significantly different from zero (*β*_0_ = 6.671, *β*_1_ = −1.248, *β*_2_ = 0.0445 with all p-values < 0.001). These coefficients imply that higher values of inferred transition probabilities ubiquitously resulted in speeding across all models (negative *β*_1_). Furthermore, post-error slowing was present (positive *β*_2_).

The bold rows in Table 1 report the log likelihood and BIC values for the fits of the HGF and the FOM on the entire data. It is evident that the FOM substantially outperformed the HGF in terms of both scores. A confusion analysis was performed to ensure the efficacy of the model selection procedure. This revealed that the log likelihood measure provided a firm ground for selection between the two model classes (confusion was less than 1% in either direction; see S1 Table). Indeed, that the FOM outperformed the HGF in terms of the log likelihood is a stronger result than the equivalent based on the BIC score, since the HGF is a far more complex model, with many more parameters.

One of the main differences between the FOM and the HGF is the use of normalised rather than unnormalised belief measures at the lowest level (i.e., exploiting the subjects’ knowledge that only a single stimulus could appear). To assess the importance of this, we considered using the count $U_{s^{t}s^{t+1}}^{t}$ rather than the normalised ${\bar{U}}_{s^{t}s^{t+1}}^{t}$ as a factor in the response component of the model. This removes the constraint that there can only be a single symbol *s*^*t*+1^. The bottom row of Table 1 shows that the unnormalised model was significantly out-performed by the normalised version, implying that subjects’ responses were indeed better described as being constrained to and by the task-relevant events.

Furthermore, the difference in log likelihood (and BIC value) between the FOM with stable beliefs (*γ* = 0) and converging beliefs (*γ* = 1) suggests that the modified belief trajectories of the latter had a significant impact on the model fit. This supports the choice of prior beliefs.

As a final test, we considered the speeding in the first and second half of the blocks (as in Fig 3c) to assess the magnitude of the observed mismatches between data and models. We compared the relative magnitudes of the differences between the HGF and the FOM and the data, and between the FOM and the data, again using permutation tests (for trials 2-3 and 3-4). We found that the HGF’s differences in speeding curves were significantly greater in magnitude to those of the FOM (*p*_2,3_ < 0.001, *p*_3,4_ = 0.03), and also to those of the data on trial 2 to 3 (*p*_2,3_ = 0.006). By contrast, comparing the FOM against the data in the same way revealed no significant difference in magnitude (all p-values ≥ 0.4).

### 3.4 Drug groups

Since the FOM was significantly more consistent with the data across all the groups of pharmacologically-manipulated subjects than the HGF (see Table 1), we compared its parameter fits with those for the placebo group to assess the effects of the drugs. Statistical tests were Bonferroni corrected for the three groups; and were corrected for differences in alertness and body weight.

We found no evidence for significant group differences between the placebo group and subjects in the DA and NA condition; thus our analyses are focused on the ACh group. In particular, we did not find this group to be significantly slower than the placebo group, and neither did the cholinergic compound significantly influence the effect of surprise on RTs (i.e., *β*_0_ and *β*_1_ were not significantly different from placebo). Instead, we found that the ACh group forgot significantly faster than the placebo group.

That the forgetting rate λ was higher for the ACh than the placebo group in the 0^th^-order contexts implies that learning was based more strongly on recent observations and reached asymptote more quickly. This result stands in contrast to the lower learning rate that was found when fitting the HGF, which implies more stability and less flexible adaptation to the environment.

### 3.5 First-order and alternating matrices

The contingencies associated with the 0^th^-order matrices are particularly simple—with one single response becoming greatly over-represented. Quantifying the performance of these models for data coming from the other matrix types (Table 2) reveals that the FOM better fit the data in all three block types, albeit with a particularly large margin for 0^th^-order matrices.

**Table 2:** Model summary split by matrix type. Number of parameters per subject, average log likelihood and BIC values for data observed in different block types for the favoured HGF and FOM fit on the entire data sequence.

Model	Parameters	average LL ×10^4^	BIC
**HGF (relaxed prior)**	10		
0^th^-order		642	52
1^sth^-order		-510	11967
alternating		75	8154
**FOM (converging beliefs, *γ* = 1)**	4		
0^th^-order		1277	-13629
1^st^-order		-390	5999
alternating		211	2075

**Fig 5:**
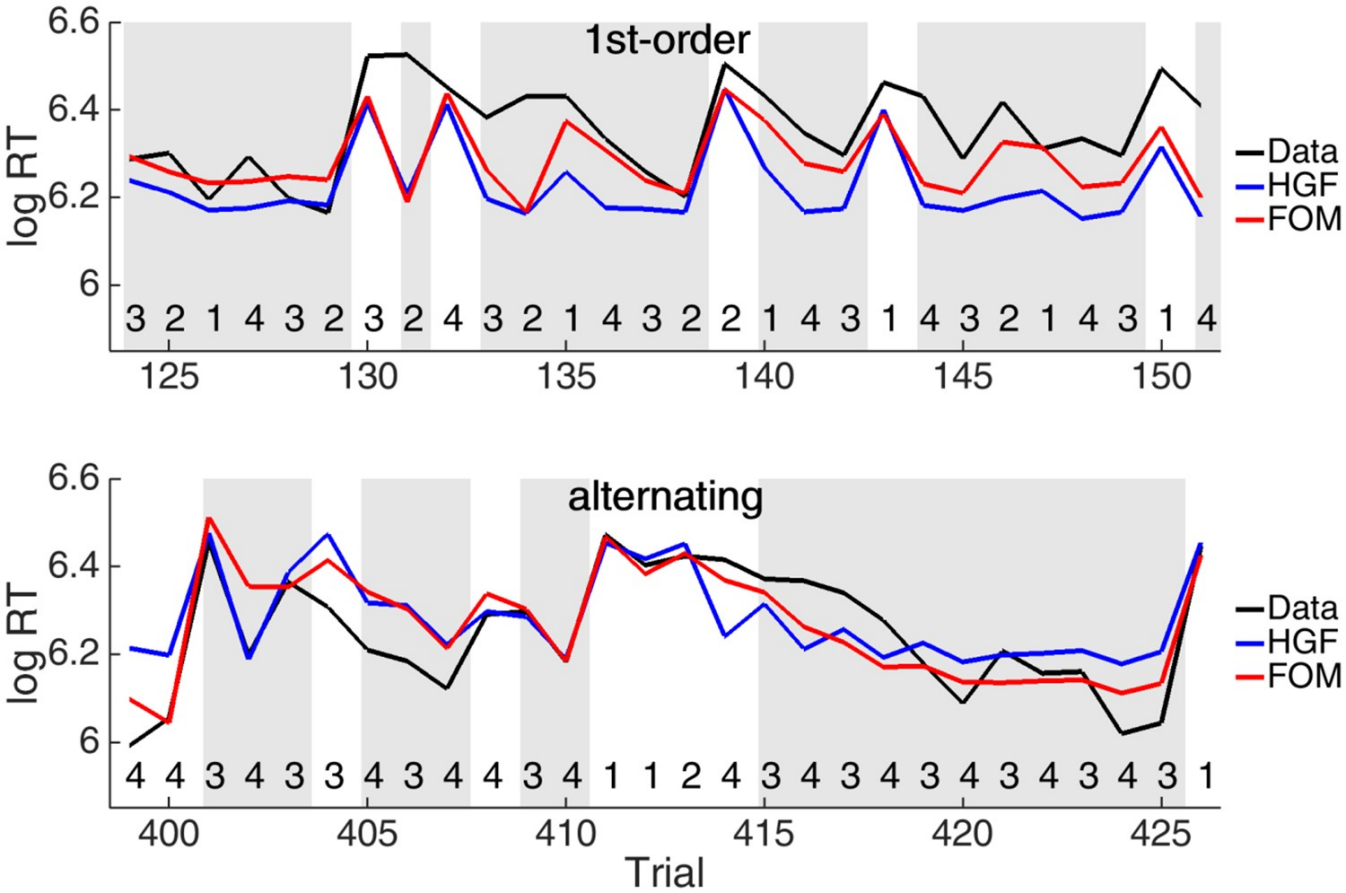
Example sequences for the 1^st^-order and alternating sequences. Shown are the data (black), and predictions for the HGF (blue) and the FOM (red) averaged over subjects in the placebo group. High probability transitions are shaded in grey.

Nevertheless, Fig 5 shows the equivalent of Fig 2 for the alternating and 1^st^-order matrix types in which different patterns of response have high probability. It is apparent that the HGF and the FOM are similarly incapable of capturing the nuances of the speeding in these cases.

**Fig 6:**
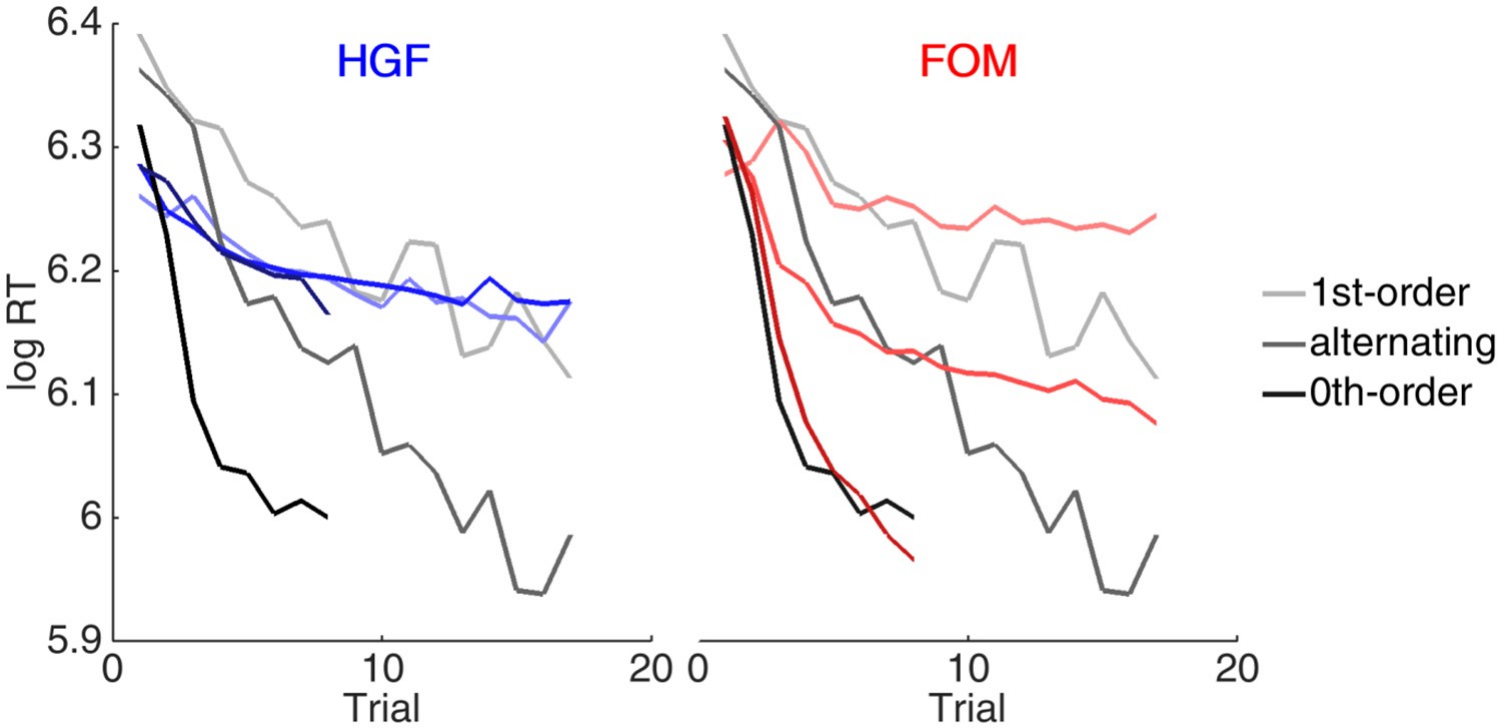
Speeding on high probability sequences for different matrix types. RTs (grey) and predictions of the HGF (blue) and FOM (red) averaged over subjects in the placebo group for all uniterrupted sequences of high probability transitions for the three matrix types.

Fig 6 provides some insight into the issues for the models, showing what happens with the high probability sequences for the other matrix types. Again, the HGF is too rigid to fit them well at all. The FOM fit the early speeding well, but then reaches an asymptote, rather than speeding further to match the subjects.

**Fig 7:**
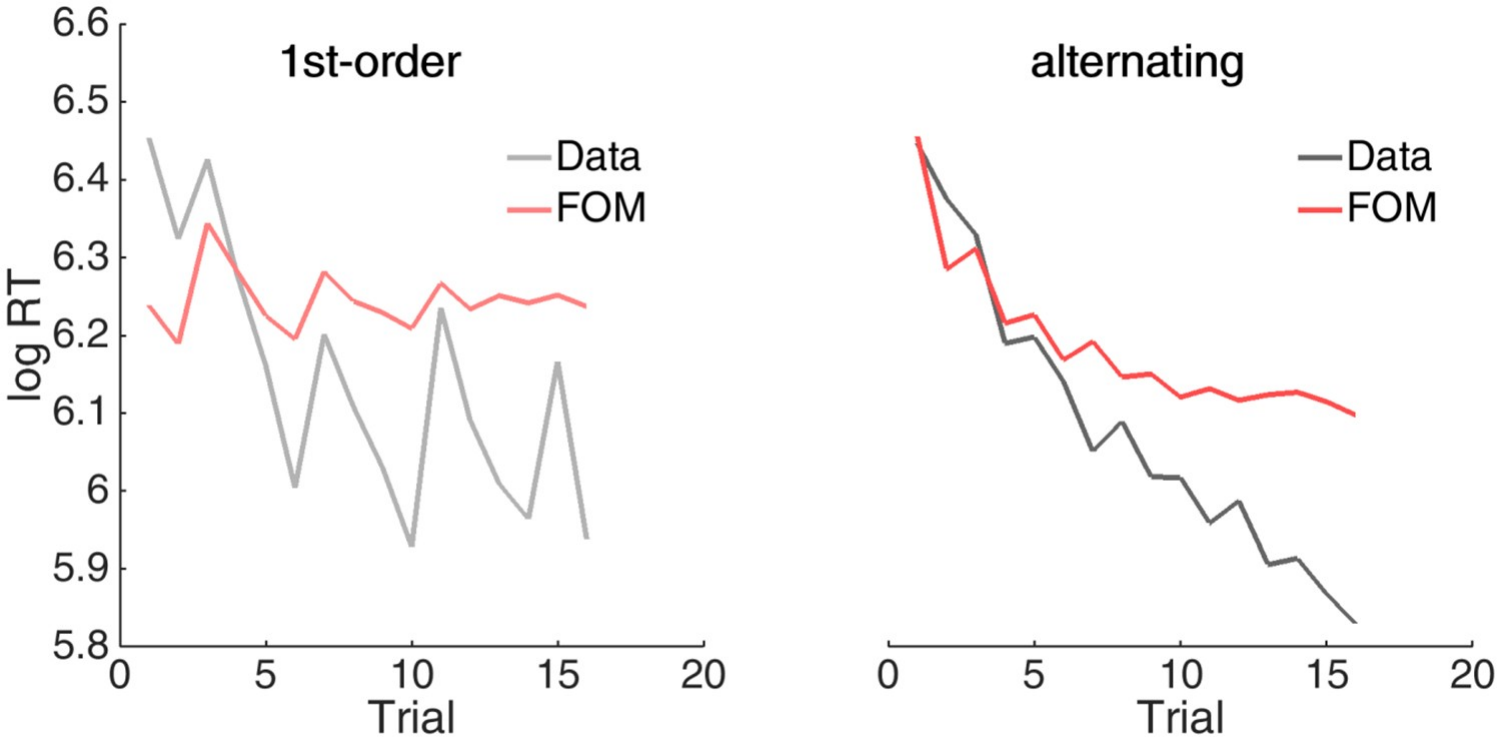
Response variability in sequences of high probability transitions. Average RTs in placebo group (grey) for a single example sequence of high probability transitions in a 1^st^-order and alternating context, and predictions of the FOM (red).

The obvious possibility is that the FOM’s simple model of forgetting may only be an approximation to the actual intricate mechanisms of adaptation and change. Fig 7 shows two examples of log RTs on particular high probability sequences that provide extra evidence for this. Here, subjects modulated their responses markedly, even though every transition was highly likely and continuous speeding might have been expected. The qualitative pattern of changes in log RTs is initially well-captured, albeit with a reduced amplitude. However, forgetting makes these expected variations flatten out over time—whereas they are, if anything, magnified in the data. One possible explanation is a form of chunking of behavioural patterns (requiring n-gram models for *n* > 1): for example, a sequence of high transition probabilities in 0^th^-order matrices is a simple repetition of one stimulus, whereas all four stimuli occur during such sequences in a 1^st^-order matrices.

Note that half of all trials belonged to 0^th^-order matrices, thus favouring fits of these contexts and thereby shifting parameter estimates to those appropriate in matrices with simple behavioural dynamics. Therefore we fitted individual FOM’s to data from the different matrix types in order to validate the parameter values for the drug groups obtained from the full model. Due to the disruption of the sequence of T’s, the FOM’s beliefs were set to be uniform at the beginning of each block. Even though the log likelihood associated with FOM’s fitted to individual transition dynamics were greater, the qualitative underfit remained (data not shown).

#### Drug groups

**Fig 8:**
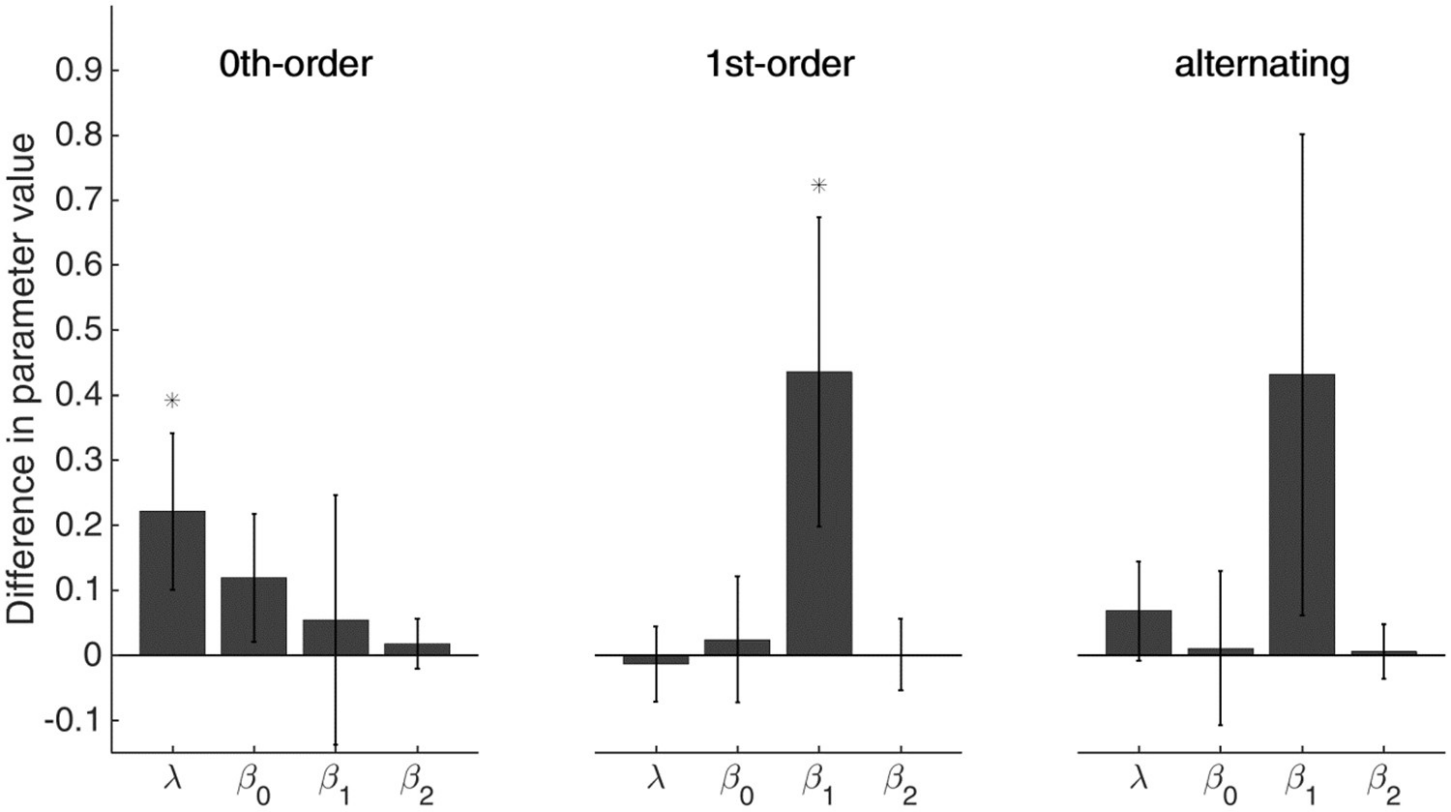
Differences in parameter values of ACh groups versus placebo for different matrix types. ACh group shows a significantly higher forgetting rate and average response time for FOM fitted on 0^th^-order matrices only, whereas FOM fitted to either 1^st^ or alternating matrices show significantly less response modulation. Error bars indicate 95%-confidence intervals; significance stars indicate significance from zero after Bonferroni correction.

We evaluated the differences between drug groups based on the parameters inferred by FOM’s fitted to data from the different matrix types. This confirmed the higher forgetting rate in case of 0^th^-order matrices and average RT for ACh group that we reported for fits of the full model (Fig 8). However, when fitting the FOM to 1^st^-order and alternating matrices, we found no significant difference in forgetting rates and average RT when comparing ACh group to placebo. Instead, we observed a more limited modulation of responses based on beliefs about transitions (i.e., a reduced magnitude of *β*_1_).

**Fig 9:**
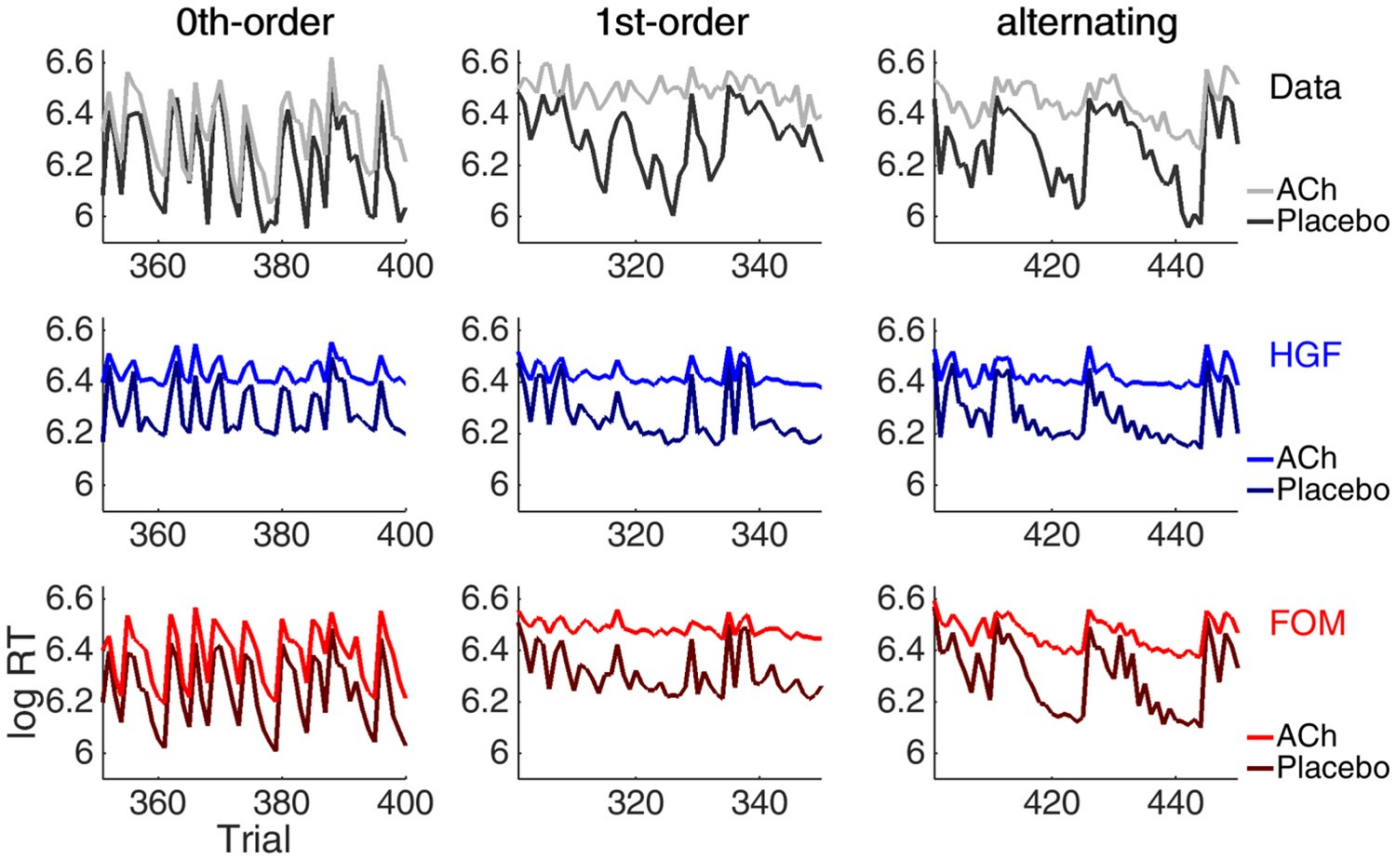
Comparison of actual and predicted responses between placebo and ACh group. Average RTs in placebo and ACh group (grey) for one example block from each of the three types and average predictions of HGF (blue) and FOM (red).

Of course, interpreting the parameter values in Fig 8 for 1^st^-order and alternating matrices is tricky because neither the HGF nor the FOM fit the data satisfyingly well. Fig 9 depicts this more starkly. It shows that the behaviour of the ACh group was strongly influenced by the complexity of the probabilistic context. While in the 0^th^-order contexts the ACh group appeared to learn to a similar degree as the placebo group, modulation of their RTs was significantly shallower in the alternating contexts and almost absent in 1^st^-order matrices. The behaviour in the 1^st^-order matrices suggests that the ACh group, instead of forgetting rapidly, might actually have learned less from their observations. In order to test this, we included an extra learning parameter in the FOM which weighted the count added to the Dirichlet parameters on each trial (i.e. allowing for weaker or stronger learning by changing the number of counts added). However, the improved fit associated with this additional parameter did not justify the increase in complexity (as assessed by the BIC score).

## 4 Discussion

We considered the characteristics of two Bayesian-inspired models of learning and their match to human behaviour on a probabilistic serial reaction time task. Both models are approximate in their characterisation of the task, reflecting the assumption that subjects did not acquire the true generative model thus rendering their behaviour sub-optimal. However, the models made different assumptions about the way that learning in a particular task can best be characterised. The HGF implements a rather generic inference method that is assumed to be able to govern learning in any task environment—it therefore faces the risk of underfitting performance in any particular task. By contrast, the FOM is more narrowly tailored to the task requirements and statistics, and so might overfit the task in the sense of not advancing the general understanding of learning.

In sum, the FOM generally accounted for the data in a more accurate and more parsimonious way. In particular, there was clear evidence that subjects were sensitive to the competition between stimuli at the output (i.e., that only one could appear at once). Furthermore, the analyses suggest that learning was characterised not only by a trialwise accumulation of evidence in favour of observed transitions but also by an additional blurring of these beliefs which we approximated by our continuous forgetting process with beliefs converging to a uniform distribution over time.

While the FOM was designed to capture those parts of the task design that were available to subjects (e.g., the expectation that there would be exactly one possible outcome per trial), forgetting is perhaps best seen as an approximation to the way that subjects dealt with the hidden hierarchical structure in the task about which they were not informed. That subjects were indeed unaware of the task structure and did not recover it during the experiment was revealed by their short window of integration that led to re-learning of transition probabilities within contextual blocks. We provided evidence that, rather than being treated hierarchically, subjects treated the observations as a mixture of short predictable sequences interrupted by random events. This led to sub-optimal behaviour under the true model of the task. These results are in line with previous findings demonstrating failings of ideal observer models in environments with changing contingencies [7–9] in which learners did not recover the task structure either. The HGF’s approximate learning scheme proved to be too inflexible to fit this data. This was reflected, for instance, in too large a window of integration. The results challenge a hope that the HGF might be a ubiquitous general-purpose inference model employed by humans. Along similar lines, it is straightforward to conceive of other tasks in which the generic algorithm implemented by the HGF is likely to deviate from human behavior, such as learning experiments in which the features of the stimuli have different degrees of predictive power [49, 50], and so may involve selective attentional mechanisms [48, 51].

The FOM captured subjects’ sub-optimal behaviour well in the simple probabilistic contexts with only one predominant outcome (the 0^th^-order matrices). This finding joins a range of work providing empirical support of behavioural models including a forgetting process [9, 37, 46–48].

Two distinct mechanism have been suggested to give rise to forgetting one in which new observations overrides past experiences, and another that creates a novel model that competes with the model acquired in the past (and with which it can be ‘switched’) (e.g. [52]). In our study we have concentrated on modelling the former type of forgetting since we found no evidence to support the mechanism of the latter (despite the design of the task explicitly encouraging this). This absence was reflected in subjects not detecting the abrupt context changes selectively, but rather displaying frequent relearning even within contextual blocks.

Thus, in our characterisation, learning and forgetting are closely related processes that are tied to each other through time. Learning about the current statistics of the world inevitably implies reduced impact of past experiences. Typically, models of this type of forgetting operate with only one time constant, however evidence suggests that multiple timescales may exist in the brain [41, 53, 54] and how they may be used to achieve flexible behaviour [7, 55]. While this is certainly an interesting possibility, we did not have enough data to investigate this further in an appropriately careful manner.

Furthermore, Fusi et al.’s [7] work suggests a potential mechanism of how forgetting could be realised on a neural level. The authors suggest that synaptic plasticity alone can give rise to many of their observed behaviours in monkeys, especially when including multiple learning rates. Other work suggests that neurons may have different temporal receptive fields which have been found predictive of behaviour [43–45].

Both the HGF and the FOM failed to describe the nature of response patterns sufficiently in more complex dynamics. This shortcoming stresses the importance of more sophisticated models of learning in order to unravel the interactions of representations and computations underlying behaviour. One possible cause is chunking, which has been demonstrated to play a significant role in motor learning of sequences [56–59]. Another possibility is that predictions have longer history dependence, i.e. not only the current stimulus predicts the next, but predictions are based on the past two trials or more (counterfactually, except around boundaries between matrices). Both such approaches would require n-gram models for *n* > 1 or perhaps learning/forgetting processes with multiple time constants. Along the latter lines is the model of Jones et al. [60] which proposes that different statistics are tracked in parallel such as stimulus base rate and probability of change. It would be interesting to modify the experimental design specifically to test these potential contributions. Moreover, the shortcomings of the FOM and HGF also limit our confidence in interpreting either model as providing complete constraints over the neural processes concerned.

However, based on its fit to the simple 0^th^-order dynamics, our model suggests that blocking ACh receptors led to increased adaptability. This was reflected in stronger unlearning of expectations over the most likely transitions and consequently stronger re-learning during predictable sequences captured by a higher forgetting rate in our model. This finding stands in contrast to prior results obtained from analyses using the HGF reporting a lower learning rate if receptors are blocked [13] and conversely, higher learning rate when ACh receptors are stimulated [31]. This discrepancy raises two questions: Why do the HGF and FOM result in opposite interpretations? How could higher adaptability during receptor blockade fit with existing theories on the neuromodulatory systems?

To the first question: the HGF’s parameter estimates are likely compromised by the restricted space of possible solutions under its model, which do not well encompass the dynamics even of the 0^th^-order dynamics.

To the second question: one possibility is that the effect comes from the interaction between different sorts of uncertainty postulated by Yu and Dayan [20, 61]. According to that account, ACh reports expected uncertainty; and other systems unexpected uncertainty (noradrenaline in that paper; the dorsal anterior cingulate cortex in [18]; a structure with projections to the noradrenergic locus coeruleus). The result of compromising expected uncertainty (ACh) is that subjects would more frequently be in a state of unexpected uncertainty, leading to the perception of even greater instability of the environment. A shorter window of integration in highly volatile environments can thus be viewed as a beneficial result of the learning mechanics.

We showed that even in the placebo group, the sequence of observations might not have been perceived in the hierarchical manner that was originally intended. Rather, the environment appeared very labile. The lack of effect following manipulations of NA could potentially have resulted from this, with an environment perceived as inherently unstable failing to trigger signals from this system. More detailed examination of the interaction between ACh and NA with a task in which the transitions are more salient would be interesting.

Of course, parameter interpretability is always limited by a model’s ability to account for the data as mechanisms might lie outside the scope of its workings, or simply because the data provides little signal. While the FOM appeared to provide a sufficiently close account of behaviour in simple dynamics, the particularly shallow response pattern of the ACh group in the complex contexts posed a significant challenge to both the models. A flat, or very noisy, learning curve might be equally well approximated by a particularly high (immediate forgetting) or low (no learning at all) learning rate, or, as reported by individually fitted FOMs, belief updating matching that found under placebo, but with a reduced weighting of top-down knowledge. In the same way, the absence of any significant overall slowing of the ACh group in the 0^th^-order blocks could be explained by the high forgetting rate, as the reduced expectations that result ultimately predict slower RTs in the linear response model. Even though an additional parameter in the FOM to explicitly adjust the speed of learning in addition to forgetting was not supported by the data, this might be a result of the model’s inability to fit higher order response features in these contexts.

The discrepancy between interpretations of parameters in the drug groups in the HGF and FOM stresses the importance of comparisons between models implementing different assumptions about the underlying process, especially when the assumptions of the models remain speculative. While it is appealing to compare results within a single over-arching modelling framework (like the HGF) since it provides a consistent ground across data sets, we have shown that this is not without challenges. Of course, inference in special-purpose models based on instructions or experience may itself be mysterious and it might actually be that subjects start off using something akin to the HGF, and build a more specific model in the light of predictive failure. However, the task here was not designed to examine this possibility.

Furthermore, our finding that a change in the prior on the response variance in the HGF had a major effect on the log likelihood and BIC score even though posterior predictions remained unchanged, draws attention to a more general issue that can arise when performing model comparison. The fact that priors are part of the statistical model that is being evaluated on the basis of goodness of fit measures, like the BIC value, stresses the importance of paying great attention to these modelling choices whose subjectivity have been put forward as criticism of the Bayesian framework ever since it came into fashion. Furthermore, it highlights the need to assess a model’s fit in direct comparison with the raw data which may reveal properties of the model that cannot be detected solely based on a one-dimensional scale.

In summary, our results show some of the potential pitfalls of using powerful, generic, inference methods to characterise human behaviour in relatively complex circumstances. They also show both the power and problems of using more specific inference methods. In terms of the latter: our subjects were more inefficient than expected in apparently ignoring a significant part of the hierarchical structure of the domain; by contrast, their behaviour suggested that they could chunk sequences of high probability actions in a way that our perceptual and response models were not readily able to capture. Nevertheless, even though interpretations of the behaviour on the full data remain speculative, our close fit to responses in the simple dynamical cases provides a window onto subjects’ processing. The results stress the importance of model comparison and provide examples of how models based on different assumptions can collectively enrich our understanding of powerful psychological mechanisms.

## Supporting information

S1 Table Confusion analysis. We performed a confusion analysis for the FOM and HGF in order to test the efficacy of our model selection procedure. For each subject in the experiment we sampled a dataset based on the parameter values estimated under either model. This resulted in 124 simulations from each model. Subsequently, we fit the FOM and HGF on both sampled datasets and recorded whether the model that generated the data was indeed supported by a higher log likelihood score. The results show that a model comparison based on the log likelihood is near perfect in telling apart FOM and HGF for the particular task of our study.
(PDF)

S1 Fig Parameter recovery. We used the same sampled datasets as in the confusion analysis to investigate the reliability of parameter estimates for the perceptual components of the FOM and HGF. For this we compared the parameters used for sampling the data with those recovered by each model. It can be obtained that the forgetting rate (λ) of the FOM was estimated accurately. Similarly, the tonic learning rate (*ω*) on the second level of the HGF was well recovered. However, the HGF’s metavolatility estimates (*ϑ*) on the third level were considerably corrupted limiting the degree to which conclusions can be drawn based on quantities from the top level of the HGF. Diagonal lines indicate optimal recovery.
(PDF)
