# Supplementary material for: Forget-me-some: General versus special purpose models in a hierarchical probabilistic task
Source: PLoS One. 2018 Oct 22;13(10):e0205974. doi: 10.1371/journal.pone.0205974 (PMC6197684; doi:10.1371/journal.pone.0205974)
Supplement: S1 Table — We performed a confusion analysis for the FOM and HGF in order to test the efficacy of our model selection procedure. For each subject in the experiment we sampled a dataset based on the parameter values estimated under either model. This resulted in 124 simulations from each model. Subsequently, we fit the FOM and HGF on both sampled datasets and recorded whether the model that generated the data was indeed supported by a higher log likelihood score. The results show that a model comparison based on the log likelihood is near perfect in telling apart FOM and HGF for the particular task of our study. (PDF) [file pone.0205974.s001.pdf]

True model \ Inferred model	FOM	HGF
**FOM**	**124**	**0**
**HGF**	**1**	**123**
