# Supplementary figures and images for: Forget-me-some: General versus special purpose models in a hierarchical probabilistic task
Source: PLoS One. 2018 Oct 22;13(10):e0205974. doi: 10.1371/journal.pone.0205974 (PMC6197684; doi:10.1371/journal.pone.0205974)

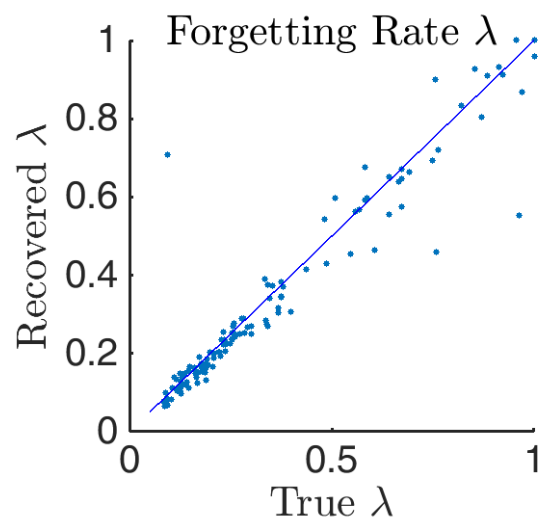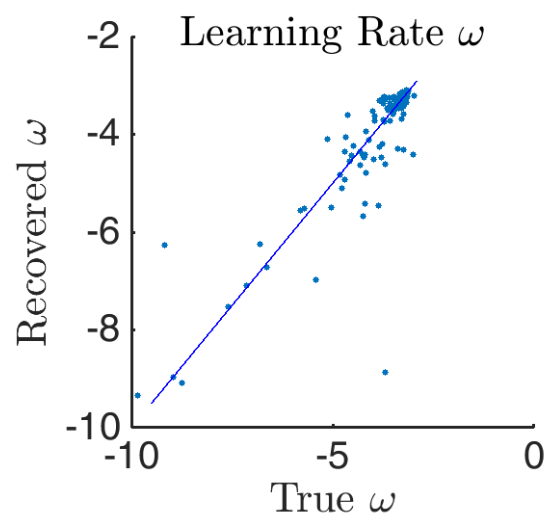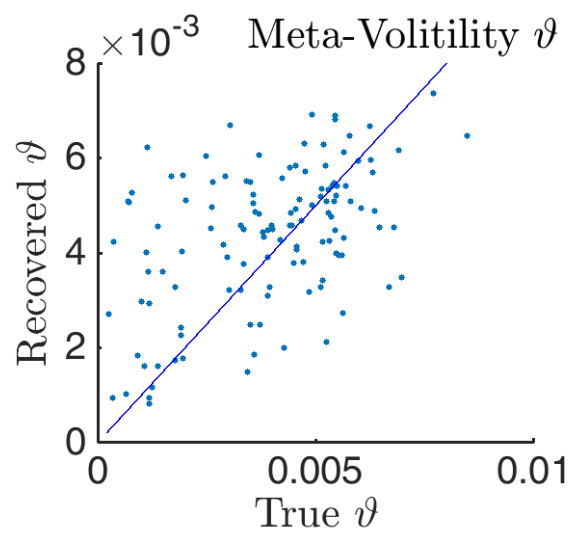

Supplement: S1 Fig — We used the same sampled datasets as in the confusion analysis to investigate the reliability of parameter estimates for the perceptual components of the FOM and HGF. For this we compared the parameters used for sampling the data with those recovered by each model. It can be obtained that the forgetting rate (λ) of the FOM was estimated accurately. Similarly, the tonic learning rate (ω) on the second level of the HGF was well recovered. However, the HGF’s metavolatility estimates (ϑ) on the third level were considerably corrupted limiting the degree to which conclusions can be drawn based on quantities from the top level of the HGF. Diagonal lines indicate optimal recovery. (PDF) [file pone.0205974.s002.pdf]
